# Vitamin D supplementation for improving bone mineral density in children

**DOI:** 10.1590/S1516-31802011000400013

**Published:** 2011-05-05

## Abstract

**BACKGROUND:**

Results of randomized controlled trials (RCTs) of vitamin D supplementation to improve bone density in children are inconsistent.

**OBJECTIVE:**

To determine the effectiveness of vitamin D supplementation for improving bone mineral density in children, whether any effect varies by sex, age or pubertal stage, the type or dose of vitamin D given or baseline vitamin D status, and if effects persist after cessation of supplementation.

**CRITERIA FOR CONSIDERING STUDIES FOR THIS REVIEW:**

We searched the Cochrane Central Register of Controlled Trials (CENTRAL Issue 3, 2009), MEDLINE (1966 to present), EMBASE (1980 to present), CINAHL (1982 to present), AMED (1985 to present) and ISI Web of Science (1945 to present) on 9 August 2009, and we handsearched key journal conference abstracts.

**SELECTION CRITERIA:**

Placebo-controlled RCTs of vitamin D supplementation for at least three months in healthy children and adolescents (aged from one month to < 20 years) with bone density outcomes.

**DATA COLLECTION AND ANALYSIS:**

Two authors screened references for inclusion, assessed risk of bias, and extracted data. We conducted meta-analyses and calculated standardized mean differences (SMD) of the percent change from baseline in outcomes in treatment and control groups. We performed subgroup analyses by sex, pubertal stage, dose of vitamin D and baseline serum vitamin D and considered these as well as compliance and allocation concealment as possible sources of heterogeneity.

**MAIN RESULTS:**

We included six RCTs (343 participants receiving placebo and 541 receiving vitamin D) for meta-analyses. Vitamin D supplementation had no statistically significant effects on total body bone mineral content (BMC), hip bone mineral density (BMD) or forearm BMD. There was a trend to a small effect on lumbar spine BMD (SMD 0.15, 95% CI −0.01 to 0.31, P = 0.07). There were no differences in effects between high and low serum vitamin D studies at any site though there was a trend towards a larger effect with low vitamin D for total body BMC (P = 0.09 for difference). In low serum vitamin D studies, significant effects on total body BMC and lumbar spine BMD were approximately equivalent to a 2.6% and 1.7 % percentage point greater change from baseline in the supplemented group.

**AUTHORS’ CONCLUSIONS:**

These results do not support vitamin D supplementation to improve bone density in healthy children with normal vitamin D levels, but suggest that supplementation of deficient children may be clinically useful. Further RCTs in deficient children are needed to confirm this.

Nutrologist. Full Professor of Pediatrics, Faculdade de Medicina da Universidade de São Paulo (FMUSP), São Paulo, Brazil.

**This is the abstract of a Cochrane Review published in the Cochrane Database of Systematic Reviews (CDSR) 2011, Issue 2, DOI:** 10.1002/14651858.CD006944.pub10 (www.thecochranelibrary.com). For full citation and authors details see reference 1.

**For Latin America and the Caribbean, the full text is freely available from:** http://cochrane.bvsalud.org/cochrane/show.php?db=reviews&mfn=4014&id=CD006944&lang=pt&dblang=&lib=COC&print=yes

**This section was edited under the responsibility of the Brazilian Cochrane Center.**

The independent commentary was written by Rubens Feferbaum.

## COMMENTS

A recent publication from the IOM-USA (Institute of Medicine of the United States)^1^ recommends that vitamin D supplementation of 400 IU/day should be provided for children < 1 year and 600 IU/day for children and adolescents aged 1-19 years, since serum vitamin D levels are believed to be below normal in many children. The effect of vitamin D is not immediate, given that osteoporosis (which is considered to be an adult disease) may begin during adolescence because of calcium and vitamin D deficits. The Cochrane review analyzes the effect of three months of vitamin D supplementation, rather than long-term. It shows that supplementation among eutrophic children is not a useful recommendation.^2^
